# Acute effects of exercise on the inflammatory state in patients with idiopathic pulmonary arterial hypertension

**DOI:** 10.1186/s12890-016-0301-6

**Published:** 2016-11-11

**Authors:** Lars Harbaum, Emilia Renk, Sara Yousef, Antonia Glatzel, Nicole Lüneburg, Jan K. Hennigs, Tim Oqueka, Hans J. Baumann, Djordje Atanackovic, Ekkehard Grünig, Rainer H. Böger, Carsten Bokemeyer, Hans Klose

**Affiliations:** 1 Center for Pulmonary Arterial Hypertension, University Medical Center Hamburg-Eppendorf, Hamburg, Germany; 2 Section Pneumology, Department of Medicine II, University Medical Center Hamburg-Eppendorf, Martinistraße 52, 20246 Hamburg, Germany; 3 Division of Hematology and Hematologic Malignancies, Huntsman Cancer Institute, University of Utah, Salt Lake City, USA; 4 Department of Stem Cell Transplantation, University Medical Centre Hamburg-Eppendorf, Hamburg, Germany; 5 Institute of Clinical Pharmacology and Toxicology, University Medical Center Hamburg-Eppendorf, Hamburg, Germany; 6 Cardiovascular Institute and Department of Pediatrics, Wall Center for Pulmonary Vascular Diseases, Stanford University School of Medicine, Stanford, USA; 7 Center for Pulmonary Hypertension, Thoraxclinic, University Hospital Heidelberg, Heidelberg, Germany

**Keywords:** Pulmonary arterial hypertension, Cytokine, Exercise, Training, Inflammation, Immunology

## Abstract

**Background:**

Exercise training positively influences exercise tolerance and functional capacity of patients with idiopathic pulmonary arterial hypertension (IPAH). However, the underlying mechanisms are unclear. We hypothesized that exercise modulates the activated inflammatory state found in IPAH patients.

**Methods:**

Single cardiopulmonary exercise testing was performed in 16 IPAH patients and 10 healthy subjects. Phenotypic characterization of peripheral blood mononuclear cells and circulating cytokines were assessed before, directly after and 1 h after exercise.

**Results:**

Before exercise testing, IPAH patients showed elevated Th2 lymphocytes, regulatory T lymphocytes, IL-6, and TNF-alpha, whilst Th1/Th17 lymphocytes and IL-4 were reduced. In IPAH patients but not in healthy subject, exercise caused an immediate relative decrease of Th17 lymphocytes and a sustained reduction of IL-1-beta and IL-6. The higher the decrease of IL-6 the higher was the peak oxygen consumption of IPAH patients.

**Conclusions:**

Exercise seems to be safe from an immune and inflammatory point of view in IPAH patients. Our results demonstrate that exercise does not aggravate the inflammatory state and seems to elicit an immune-modulating effect in IPAH patients.

## Background

Pulmonary arterial hypertension (PAH) is a devastating, progressive vascular disease characterized by elevated pulmonary vascular resistance and right heart failure. Idiopathic PAH (IPAH) is diagnosed when no known condition or exposure associated with PAH is detectable. In pulmonary arteries of patients with IPAH a prominent peri-vascular inflammation including tertiary (ectopic) lymphoid follicles was frequently observed next to endothelial dysfunction, *in-situ* thrombosis, loss of small vessels and occlusive vascular remodeling [18, 23, 24]. The local inflammatory process is accompanied by an altered systemic inflammatory status (or vice versa). Levels of circulating cytokines and inflammatory cells were found to be altered in patients with IPAH [5, 12, 18, 22, 25]. Peripheral regulatory T lymphocytes and interleukin (IL)-17 producing Th lymphocytes were found to be increased and C-C chemokine receptor (CCR) 6^+^ T lymphocytes depleted in peripheral blood samples taken from IPAH patients [12, 18, 25]. Interestingly, it has been shown that the chemokine receptors CCR4 and CXCR3 distinguish two stable subsets of IL-17-producing cells within the CCR6+ compartment, wherein CCR6+/CCR4-/CXCR3+ cells produced IL-17 and INF-gamma (i.e. Th1/Th17) [1]. The emerging focus on inflammation lends itself to a whole new approach in understanding the disease and to a new perspective of treating IPAH.

Physical exercise has an anti-inflammatory effect in chronic cardiovascular diseases by amelioration of chronic inflammation [16, 20]. Contracting skeletal muscle cells produce cytokines such as IL-1ra, IL-6 and IL-10 [10]. Immune-modulation and down-regulation of the chronic inflammation seem possible due to repeated short-lasting alterations in cytokine levels by exercise [10]. On the other hand pro-inflammatory cytokines diminish in the long term due to the reduction of the visceral fat mass [10]. In advanced cardiopulmonary diseases such as IPAH, however, cachexia and physical degeneration may be present. Patients with IPAH who underwent supervised exercise training reached higher levels of physical activity, had decreased fatigue severity, showed improved 6-min walk distance (6MWD), cardiopulmonary function such as improved peak oxygen consumption and patient-reported quality of life [6, 8, 9]. Thus, it is recommended to implement controlled exercise training and rehabilitation in PAH patients’ care taking [3, 8, 9, 11, 27]. However, the underlying mechanisms leading to the improvement of symptoms, exercise, and functional capacity are not clear [9].

In this study, we present the first evidence for an exercise-induced immune response in patients with IPAH. The single bout of exercise in IPAH patients seems to be safe from an immune and inflammatory point of view and we demonstrate that the immune system reacts differently in patients with IPAH as compared to healthy control subjects.

## Methods

### Study subjects and procedure

Patients were recruited from the Center of Pulmonary Arterial Hypertension at the University Medical Center Hamburg-Eppendorf with the following inclusion criteria: (1) prevalent diagnosis of IPAH in accordance with the current diagnostic recommendations and classification [9], (2) >18 years of age, (3) World Health Organization functional class (WHO FC) II-III, (4) no evidence for current or past malignant or autoimmune diseases, (5) without exercise limitation due to an orthopedic reason, and (6) without current infectious disease. Healthy subjects underwent echocardiography to rule out signs of right or left heart failure and were matched with regard to age, gender and body mass index (BMI). Cardiopulmonary exercise testing (CPET) was performed as symptom-limited incremental cycling exercise according to the ERS task force (2007) [17]. The rate of workload increase was 10 Watt/min. The anaerobic threshold (AT) was indirectly measured as the point when the VCO_2_ − VO_2_ slope abruptly increases. The peak oxygen consumption (Peak VO_2_/kg [ml/min/kg]) was used for analysis because it represents the most widely used CPET parameter for therapeutic decision making in PAH according to current recommendations [9]. No interruption of drug intake was required and the time between exercise testing and drug intake (PAH targeted therapy) was 2–4 h. The following assessments were obtained from all study subjects at the day of CPET: 6MWD was obtained using a standardized protocol in accordance with the American Thoracic Society statement (Measuring the distance patients can quickly walk on a flat, hard surface 30 m hallway in a period of 6 min. Patients are allowed to stop and rest during the test), functional class according to the modified WHO classification was assessed by the attending physician, N-terminal pro-hormone of brain natriuretic peptide (NT-proBNP) concentrations were measured using a commercially available assay (Dimension Vista, Siemens Healthcare Diagnostics, IL, USA; Catalogue No. K6423A), echocardiography and pulmonary function test (PFT) including spirometry, body plethysmography and gas dilution.

### Differential white blood cell count and fluorescence-activated cell sorting (FACS)

**Fig. 1:**
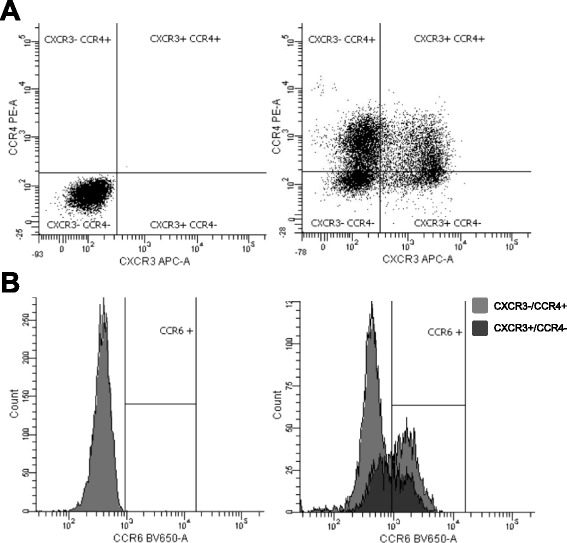
Fluorescence-activated cell sorting of T helper (H) lymphocytes (CD3+/CD4+): The surface marker CXCR3 and CCR4 differentiated four subsets of T_H_ lymphocytes after controlling for specificity of primary antibodies using the appropriate isotype control (**a**). CXCR3-/CCR4+ and CXCR3+/CCR4- T_H_ lymphocytes were characterised as CCR6+ or CCR6-, after controlling for specificity of primary antibody (**b**). APC = Allophycocyanin. BV = Brilliant Violet. CD = Cluster of differentiation. CCR = Chemokine (C-C motif) receptor. CXCR = Chemokine (C-X-C motif) receptor. PE = R-Phycoerythrin

Peripheral venous whole blood samples were taken at rest before, directly after and 1 h after CPET. Differential blood count was obtained (Coulter Ac-T diff 2, Beckmann Coulter, Krefeld, Germany) for absolute and relative numbers of leukocytes, neutrophils, lymphocytes and monocytes. Immunophenotyping was performed on whole blood samples with multicolor (4–6 colors) flow cytometry (LSR Fortessa, BD Bioscience, NJ, USA). In brief, 100 μl blood samples were incubated with staining buffer containing a pre-titrated concentration of fluorescent monoclonal antibodies or with immunoglobulin (Ig) isotype-matched controls, respectively (all BioLegend, CA, USA). Flurochromes were Alexa fluor 700 (anti-CD3), allophycocyanin (anti-CD8, -CD45, -CD127, -CD183), brilliant Violet (BV) 421 (anti-TCRγδ), BV605 (anti-CD56), BV650 (anti-CD196), fluorescein isothiocyanate (anit-CD3, -CD14), pacific blue (anti-CD19), R-phycoerythrin (anti-CD4, -CD16, -CD194) and peridinin-chlorophyll-protein complex (anti-CD4). Compensation has been performed computationally before analysis. Erythrocytes were lysed (RBC Lysis/Fixation solution, BioLegend, CA, USA), samples were washed twice with buffer solution (phosphate-buffered saline, 0.1 % bovine serum and 0.02 % NaN_3_), pelleted by centrifugation (350 × g for 5 min) and stored in buffer solution in the dark at 4 °C until analysis. Flow cytometric analysis was performed immediatly after blood sampling. T and B lymphocytes were gated by expression of CD3 or CD19 out of CD45^+^/CD16^low^/CD14^−^ cells (Table 1). T lymphocyte phenotypes were gated by size exclusion, CD3^+^ and subsequent expression of CD4^+^/CD8^−^ (T helper), CD4^−^/CD8^−^ (cytotoxic T) or CD4^−^/CD8^−^/TCRγδ^+^ (TCRγδ) (Table 1). T helper lymphocytes were gated by size exclusion, CD3+/CD4+ and subsequent expression of surface chemokine receptors, which allowed for the distinction of four different memory T helper lymphocytes, CXCR3^+^/CCR4^−^/CCR6^−^ (Th1), CXCR3^−^/CCR4^+^/CCR6^−^ (Th2), CXCR3^−^/CCR4^+^/CCR6^+^ (Th17) and CXCR3^+^/CCR4^−^/CCR6^+^ (Th1/Th17), as described previously (Fig. 1 and Table 1) [1, 4]. Regulatory T lymphocytes (Treg) were identified as T helper cells expressing high levels of CD25 and low levels of CD127 (Table 1). Natural killer cells were gated by expression of CD45/CD56 and subsequent expression of CD3 (Table 1). The flow cytometry data were collected using FACS-Diva software (BD Bioscience).

**Table 1:** Circulating peripheral blood mononuclear cells quantified by flow cytometry

Tube	Cell subset	Immunophenotypes	
		Paternal cells	Derivative cells
1	Natural killer T cells	CD45^+^/CD56^+^	CD3^+^
	Natural killer cells		CD3^−^
	B lymphocytes	CD45^+^/CD16^low^/CD14^−^	CD19^+^
	T lymphocytes		CD3^+^
2	T helper lymphocytes	CD3^+^	CD4^+^/CD8^−^
	Cytotoxic T lymphocytes		CD4^−^/CD8^+^
	TCRγδ lymphocytes		CD4^−^/CD8^−^/TCRγδ^+^
3	T_H_1 lymphocytes	CD3^+^/CD4^+^	CXCR3^+^/CCR4^−^/CCR6^−^
	T_H_1/T_H_17 lymphocytes		CXCR3^+^/CCR4^−^/CCR6^+^
	T_H_17 lymphocytes		CXCR3^−^/CCR4^+^/CCR6^+^
	T_H_2 lymphocytes		CXCR3^−^/CCR4^+^/CCR6^−^
4	Regulatory T lymphocytes	CD3^+^/CD4^+^	CD25^high^/CD127^low^

*CD* cluster of differentiation, *CCR* C-C motif receptor, *TCR* T cell receptor, *T*_*H*_ T helper lymphocytes

### Enzyme linked immunosorbent assay (ELISA) and magnetic bead-based multiplex immunoassay

Serum samples were stored at −80 °C and thawed only for analyses. Serum concentrations of IL-6 and IL-17A were measured using human IL-6 ELISA kit (Pierce Biotechnology, IL, USA) and Biosource IL-17 Cytoscreen (Life Technologies, CA, USA) following the manufacturer’s instructions. ELISA standards and samples were run in duplicate. The sensitivity of the assays was 1 pg/l for IL-6 and 2 pg/l for IL-17A. The optical density (450 nm) of each sample was determined using a microplate reader (Sunrise, Männedorf, Switzerland). Serum concentrations of IL-1-beta, IL-1ra, IL-2, IL-4, IL-10, IL-12p70, IL-13 and TNF-alpha were analyzed using a magnetic bead-based multiplex assay solution (Bio-Plex Pro TM Human Cytokine Standard 27-Plex Group I, Bio-Rad Laboratories, CA, USA) according to manufacturer’s protocol. In short, standards and samples were diluted 1:4 in buffer and added to microplates containing assay beads in duplicate. The data were collected using the Bio-Plex 200 suspension array system (Bio-Rad Laboratories).

### Statistical analysis

The differences between patients and healthy controls were compared using the unpaired Student’s t test in parametric data and the Chi-square-test in nominal data. Correlations were assessed by Pearson’s coefficient. One-way repeated measures analysis of variance (rANOVA) was conducted to analyze the difference within patients or healthy subjects, respectively, over time (i.e. before, direct after and 1 h after CPET). Two-way repeated measures rANOVA was conducted to analyze the differences between patients and healthy subjects over time. P-values as result of rANOVA are presented following Wilks’ Lambda. All reported p-values were two-sided and considered significant at levels <0.05. All statistical calculations were performed using SPSS statistics version 20 software (IBM, NY, USA).

## Results

### Baseline characteristics of study subjects

Hemodynamic parameters of 16 IPAH patients were mean pulmonary arterial pressure (mPAP) of 51 ± 19 mm Hg, pulmonary vascular resistance (PVR) of 1065 ± 809 dyne*s*cm^5^, pulmonary artery wedge pressure (PAWP) of 10 ± 3 mm Hg, right arterial pressure (RAP) of 7 ± 5 mm Hg and cardiac index (CI) of 2.18 ± 0.6 L/min*m^2^. Mean time between right heart catheterization and study inclusion was 18 ± 10 months. At time of study, six IPAH patients were in WHO FC II and 10 in WHO FC III. Fifteen patients were treated with an endothelin-receptor antagonist (ERA), 14 patients with a phosphodiesterase type 5 inhibitor (PDE5-I), 1 patient with a soluble guanylate cyclase stimulator (sGC-S) and 3 patients with a prostacyclin analog. Only one patient was treated with monotherapy and none of the patients received long-term oxygen treatment.

Matching of healthy control subjects to patients with IPAH was achieved for age, gender and BMI as intended. All tested CPET parameters were significantly reduced in IPAH patients compared to healthy control subjects. FEV_1_ and FEV_1_/FVC were significantly decreased in IPAH patients yet still within normal ranges. Levels of c-reactive protein (CRP) were < 5 mg/dl in all study subjects. Table 2 summarizes the baseline characteristics of the study subjects.

**Table 2:** Characteristics of patients with idiopathic pulmonary arterial hypertension (IPAH) and healthy subjects

Parameter	Groups		*P-value*
	IPAH	Healthy subjects	
	*n* = 16	*n* = 10	
Age [yr]	58 ± 16	58 ± 15	0.97
Gender female/male [n]	12/4	6/4	0.42^a^
BMI [kg/m^2^]	25 ± 6	24 ± 3	0.75
6MWD [m]	490 ± 100	638 ± 110	0.002
NT-proBNP [ng/l]	667 ± 594	68 ± 27	0.004
Cardiopulmonary exercise test (CPET)			
Workload max [W]	73 ± 32	130 ± 46	0.005
Peak VO_2_/kg [ml/min/kg]	15 ± 4	23 ± 5	0.001
Peak VO_2_ [l/min]	1.1 ± 0.4	1.7 ± 0.6	0.001
EqCO_2_ at AT [l/min/l/min]	47 ± 7.6	32 ± 2.2	<0.001
VO_2_ at AT [l/min]	0.8 ± 0.2	1.2 ± 0.2	0.002
HR rest/max [/min]	78 ± 12/129 ± 28	77 ± 11/138 ± 18	0.90/0.35
BP systolic rest/max [mm Hg]	102 ± 20/149 ± 30	118 ± 22/179 ± 22	0.06/0.01
BP diastolic rest/max [mm Hg]	69 ± 12/80 ± 12	75 ± 10/81 ± 13	0.25/0.60
Pulmonary function test (PFT)			
FEV_1_ % predicted [%]	78 ± 13	94 ± 8	0.002
FVC % predicted [%]	87 ± 13	95 ± 8	0.11
FEV_1_/FVC [%]	86 ± 11	102 ± 7	<0.001
DLCO % predicted [%]	76 ± 15	-	-

Data are presented as mean ± SD or numbers. P-values are calculated by Students’ t-test and ^a^Chi-Square-test. *6MWD* 6-min walk distance, *AT* Anaerobic threshold, *BMI* Body mass index, *BP* Blood pressure, *EqCO2* Respiratory equivalent for carbon dioxide, *FEV1* Forced expiratory volume in 1 s, *FVC* Forced vital capacity, *HR* Heart rate, *IPAH* Idiopathic pulmonary arterial hypertension, *NT-proBNP* N-terminal prohormone of brain natriuretic peptide, *VO2/kg* Maximum oxygen consumption per kilogram

### Before exercise patients with IPAH showed a differing composition of the T helper lymphocyte compartment and altered levels of circulating cytokines

**Fig. 2:**
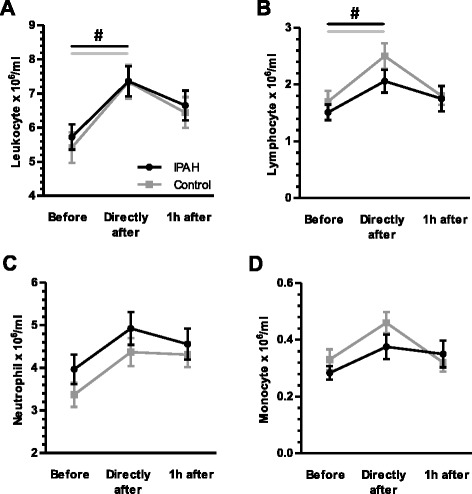
Exercise-induced changes of leukocytes, lymphocytes, neutrophils and monocytes: Exercise provoked significantly a transient elevation of leukocytes and lymphocytes in patients with idiopathic pulmonary arterial hypertension (IPAH) and healthy subjects (**a** and **b**). No differences occurred between groups. Whilst neutrophils and monocytes demonstrated only a non-significant trend towards a transients elevation (**c** and **d**). ^#^ *p* < 0.05 (rANOVA)

There was no significant difference in absolute levels of leukocytes, lymphocytes, neutrophils and monocytes before exercise between IPAH patients and control subjects (Fig. 2). Within the CD4^+^ T helper lymphocyte compartment patients with IPAH showed elevated subsets of Th2 and regulatory T cells, while the subset of Th1/Th17 lymphocytes was reduced as compared to healthy subjects and as indicated by relative and absolute cell counts (all *p* < 0.05; two-way rANOVA; Figs. 4 and 5). Prior to exercise, at rest, patients with IPAH demonstrated elevated levels of IL-6 and TNF-alpha, whilst level of IL-4 was reduced (all *p* < 0.05; two-way ANOVA; Fig. 6).

### Exercise caused a general increase in leukocytes and lymphocytes

**Fig. 3:**
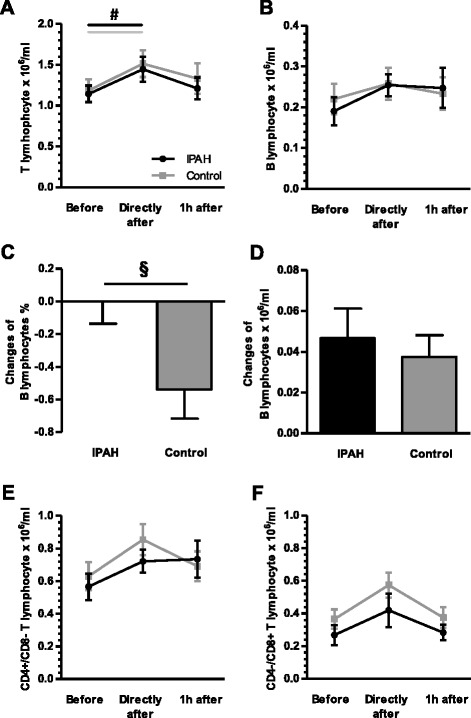
Exercise-induced changes in lymphocyte subsets: Exercise caused a transient significant elevation of T lymphocytes in patients with idiopathic pulmonary arterial hypertension (IPAH) and healthy subjects (**a**), but not in B lymphocytes (**b**). In the latter, however, exercise led to a relative reduction in healthy subjects but in IPAH patients, which was not observed for absolute numbers of B lymphocytes (**c** and **d**). Exercise provoked by a non-significant trend an elevation of CD4+/CD8- T helper and CD4-/CD8+ cytotoxic T lymphocytes in patients with IPAH and healthy subjects (**e** and **f**). CD = Cluster of differentiation. ^§^ *p* < 0.05 (t-test); ^#^ *p* < 0.05 (rANOVA)

Overall, exercise provoked a significant, transient elevation of absolute numbers of leukocytes, lymphocytes in patients with IPAH as well as in healthy subjects (all *p* < 0.001; one-way rANOVA; Fig. 2a-b). Given that no difference occurred between patients and healthy subjects, two-way rANOVA revealed the number of leukocytes and lymphocytes significantly depended on relation to exercise (*p* < 0.001 and *p* = 0.005), but not on IPAH (*p* = 0.63 and *p* = 0.28). The general increase of lymphocytes in patients with IPAH and healthy was also found for T lymphocytes (both *p* < 0.01; one-way rANOVA), and by a non-significant trend for B lymphocytes, T helper lymphocytes and cytotoxic T lymphocytes (Fig. 3a-b and e-f). Interestingly, exercise led to a relative reduction of B lymphocytes in healthy subjects but not in patients with IPAH (Fig. 3b-d). However, exercise did not influence the level of TCR-γδ lymphocytes, nor natural killer cells or natural killer T lymphocytes (data not shown).

### Exercise caused a relative reduction of Th17 lymphocytes in patients with IPAH

**Fig. 4:**
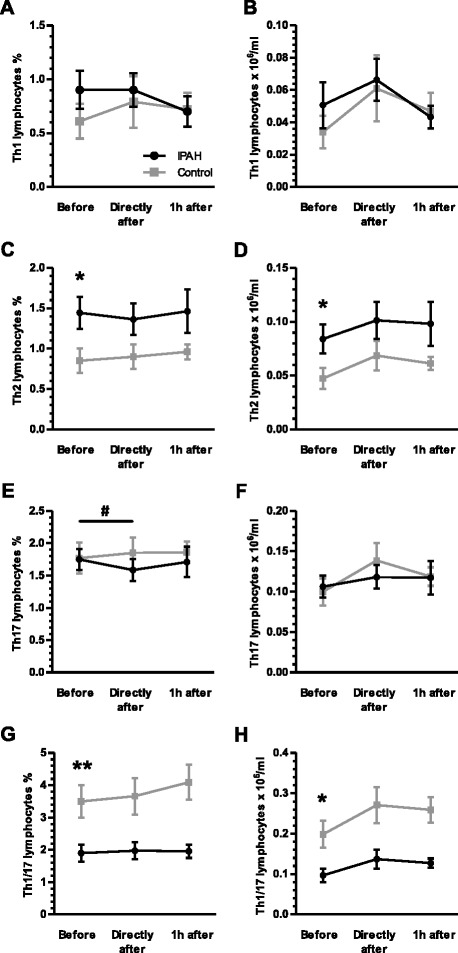
Exercise-induced changes of T helper (h) lymphocyte subsets: No significant alterations in regard of relative or absolute numbers of CXCR3+/CCR4-/CCR6- (Th1) lymphocytes occurred (**a** and **b**). Prior to exercise patients with idiopathic pulmonary arterial hypertension (IPAH) showed significantly elevated relative and absolute subsets of CXCR3-/CCR4+/CCR6- (Th2) cells (**c** and **d**) and significantly decreased levels of CXCR3+/CCR4-/CCR6+ (Th1/Th17) cells (**g** and **h**) as compared to healthy subjects. Exercise did not significantly change these subsets (**c**, **d**, **g** and **h**). However, exercise induced a relative but not absolute transients reduction of CXCR3-/CCR4+/CCR6+ (Th17) lymphocytes in patients with IPAH (**e** and **f**). CCR = Chemokine (C-C motif) receptor. CXCR = Chemokine (C-X-C motif) receptor. *p < 0.05 (rANOVA); **p < 0.01 (rANOVA); ^#^ *p* < 0.05 (rANOVA)

**Fig. 5:**
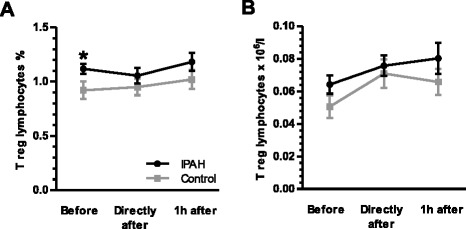
Exercise-induced changes of regulatory T helper lymphocytes: Prior to exercise relative, but not absolute, level of CD25high/CD127low (T regs) were significantly elevated in patients with idiopathic pulmonary arterial hypertension (IPAH) as compared to healthy subjects (**a** and **b**). Exercise, however, elicited no significant effect. CD = Cluster of differentiation. *p < 0.05 (rANOVA)

Within the T helper lymphocyte compartment throughout exercise no significant differences occurred regarding the subsets of Th1, Th2, Th1/Th17 and regulatory T lymphocytes comparing patients with IPAH and healthy subjects (Figs. 4 and 5). Exercise, however, caused a relative reduction of Th17 lymphocytes in patients with IPAH, but not in healthy subjects (*p* = 0.049; one-way rANOVA; Fig. 4). However, the differences appeared to be very small with significant overlap in standard deviation and no differences were observed in regard of absolute numbers of Th17 cells (Fig. 4).

### Exercise caused a reduction of IL-6 and IL-1-beta in patients with IPAH

**Fig. 6:**
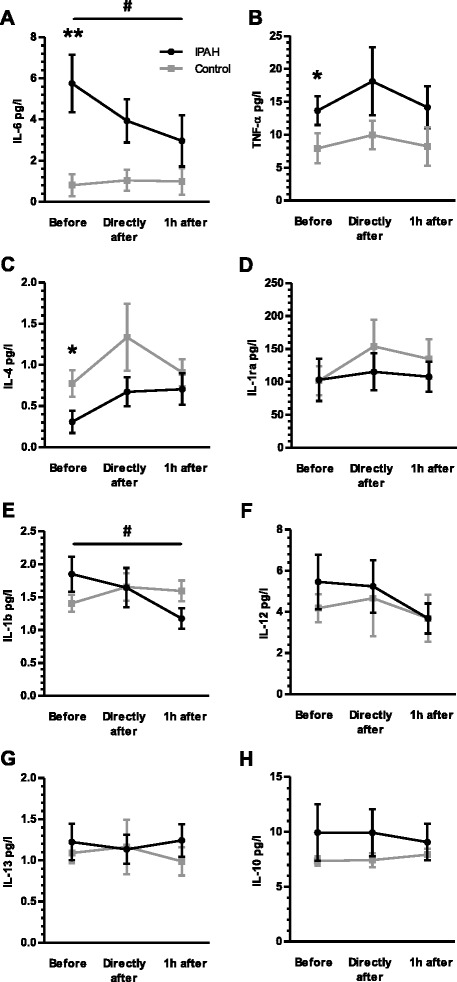
Exercise-induced changes of circulating cytokines: Prior to exercise patients with idiopathic pulmonary arterial hypertension (IPAH) showed significantly elevated levels of interleukin (IL)-6 (**a**) and tumor necrosis factor (TNF)-alpha (**b**), whilst level of IL-4 (**c**) were significantly reduced as compared to healthy subjects. No significant differences, occurred regarding IL-1ra (**d**), IL-1-beta (**e**), IL-12 (**f**), IL-13 (**g**) or IL-10 (**h**). Exercise caused a sustained reduction of the IL-6 and IL-1-beta level in IPAH patients (rANOVA; **a** and **e**). *p < 0.05 (rANOVA); **p < 0.01 (rANOVA); ^#^ *p* < 0.05 (rANOVA)

**Fig. 7:**
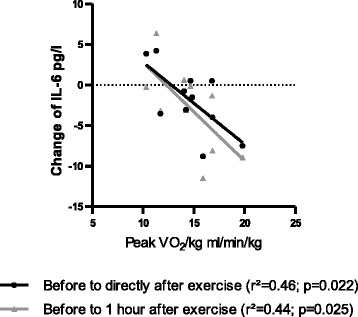
Correlation with peak oxygen consumption (peak VO2/kg): Exercise-induced changes of circulating interleukin (IL) 6 level correlate with the peak oxygen consumption in patients with idiopathic pulmonary arterial hypertension (IPAH)

Exercise caused a sustained reduction of the IL-6 level in patients with IPAH but not in healthy subjects (*p* = 0.022; one-way rANOVA; Fig. 6a). Since healthy subjects showed only a marginal increase in IL-6 following exercise, two-way rANOVA revealed that the level of IL-6 significantly depended on IPAH (*p* = 0.009), but not on relation to exercise (*p* = 0.094). In addition, the immediate and 1 h lasting reduction of the IL-6 level correlated significantly with the peak VO_2_/kg in patients with IPAH (*p* = 0.022 and *p* = 0.025; Fig. 7). Furthermore, exercise caused a reduction of the IL-1-beta level in patients with IPAH but not healthy subjects (*p* = 0.022; one-way rANOVA; Fig. 6e). Herein, two-way rANOVA revealed no significant dependence. The change of IL-6 and IL-1-beta was not significantly related to the time to exhaustion in the CPET and findings were not influenced by change in hemoconcentration (data not shown). Exercise did not influence differently the level of TNF-alpha, IL-1ra, IL-12p70, IL-13 or IL-10 in patients with IPAH as compared to healthy subjects (Fig. 6). The cytokines IL-17 and IL-2 were detectable in only 1 out of 16 patients with IPAH, respectively.

## Discussion

The development of a chronic systemic inflammatory state has been demonstrated repeatedly in patients with IPAH. This inflammatory state is indicated by elevated levels of circulating inflammation markers, such as IL-6, and altered composition of peripheral blood mononuclear cells (PBMC), such as regulatory T lymphocytes or CCR6^+^ T lymphocytes [5, 18, 22, 25]. In accordance, we found, prior to exercise, an elevated level of IL-6 and altered composition of the CD4^+^ T lymphocyte subsets in the peripheral blood of patients with IPAH. Within the CD4^+^ T lymphocyte compartment Th1/Th17 lymphocytes were depleted in blood samples from patients with IPAH and Th2 as well as Treg lymphocytes were elevated. While Th17 lymphocytes were not altered prior to exercise.

The depletion of the CXCR3^+^/CCR4^−^/CCR6^+^ (Th1/Th17) together with the observed increased expression of CCL20, the ligand of CCR6, in lungs explanted from patients with IPAH provide some evidence of the recruitment of specific T helper lymphocytes to IPAH lungs [18]. The CXCL10, ligand of CXCR3, was also found to be elevated in IPAH lungs and blood samples, which might favor the recruitment of CXCR3^+^ effector T cells [13, 28]. These inflammatory processes might therefore be inextricably linked to a specific (presumably a Th1/Th17-predominant) (peri-) vascular inflammation found in IPAH.

According to our data, exercise seemed to elicit a specific modulation of the circulating T lymphocyte subsets in patients with IPAH, a modulation of the chronic systemic inflammatory state. Exercise may shift the imbalance of the inflammatory status towards equilibrium or excess of the anti-inflammatory (or immune-regulatory) activity. A single bout of cycling exercise caused an immediate relative decrease of Th17 lymphocytes in patients compared to healthy subjects. However, the effect was small-sized and prior to exercise no differences in Th17 levels were observed. An increased Th17 immune polarization as part of the dysregulated immune response in patients with IPAH has recently been demonstrated [12]. This study further substantiate autoimmunity as contribution to the development or progression of IPAH [12].

Environmental stress such as exercise could induce a redistribution of T lymphocytes within lymphoid and non-lymphoid organs. A uniform response pattern seems to exist with a decrease in lymphocyte numbers in the spleen, accompanied by an increase in lymphocyte numbers in the lung, bone marrow and Peyer’s patches [2, 15, 26]. Accordingly, we observed a transient increase in the circulating lymphocyte numbers and also leukocyte numbers following exercise. The distribution of circulating lymphocytes is tightly controlled by their ability to interact with endothelial cells at different anatomical sites, which enables cell trafficking and defines the kinetics of immune regulation [15]. Exercise might alter the site- and disease-specific repertoire of adhesion molecules on lymphocytes and, on the other hand, the expression of corresponding ligands on endothelial cells. This might be elicited by exercise-induced alterations in circulating cytokine levels [14, 19, 29]. Moderate exercise might, for instance, result in a Th1/Th2 imbalance in favor of Th1 in healthy subjects [29]. Moreover, the addition of lymphocytes from the marginated pool into the circulation in response to exercise may influence lymphocyte redistribution by itself, since the mobilized cells may have different functional abilities compared to those already in the circulation [26]. However, redistribution of lymphocytes has mostly been demonstrated for multiple exercise training or endurance exercise protocols. Another mechanism for the modulation of lymphocyte populations might be the differentiation of lymphocytes. A rapid modulation following single exercise training might result from an (trans-) differentiation of lymphocyte from precursor cells, which could be mediated by circulating cytokines.

A high level of the cytokine IL-6 (4^th^ quartile), amongst other cytokines such as IL-2, IL-8, IL-10, and IL-12p70, were associated with poor overall survival in patients with IPAH or hereditary PAH (HPAH) [22]. The systemic inflammatory response may reflect disease activity. We observed a sustained reduction of circulating IL-1-beta and IL-6 following exercise in patients with IPAH. In contrary to this finding several studies have reported an exercise-induced release of IL-6 by contracting muscle cells in health and disease state. It is believed that the exercise-induced secretion of IL-6 to be a transcriptional event in skeletal muscle cells and was observed to be depended on exercise intensity and duration [7]. However, since exercise duration was short in our study, this mechanism was unlikely to influence post-exercise level of IL-6 in patients with IPAH. Thus further mechanisms such as internalization following receptor binding (e.g. in the process of lymphocyte differentiation or in endothelial cells) might account for the observed reduction. Furthermore, we cannot rule out that pharmacological treatment influences the exercise-induced IL-6 response, since all patients were on PAH targeted drugs. An influence of drug intake such as indomethacin on exercise-induced IL-6 response has been shown previously [7]. IL-6 has been linked to the pathogenesis of PAH. Administration of IL-6 is sufficient to cause pulmonary vascular remodeling in rodents and its over-expression spontaneously caused experimental pulmonary hypertension (PH) [21]. Less is known in regard of the pathogenic role of IL-1-beta in the setting of IPAH. The level seemed to be elevated in patients with IPAH or HPAH, but was not associated with survival [21, 22].

With the present explorative study, we looked into short-term, immediate effects of exercise on the systemic inflammatory state in a limited number of patients with IPAH. The relative small sample size, the short-term measuring points and the lack of bioelectrical indices such as visceral fat percentage are limitations to the study. With regard to a long-term effect it would be of great interest to evaluate the effect of a controlled, supervised training on the systemic inflammatory state in patients with (I)PAH. Our results provide first evidence for further investigations of this topic.

## Conclusion

We demonstrate a link between the immune system and physical exercise in patients with IPAH. Exercise seems to elicit a modulation or redistribution of the circulating T lymphocyte subsets and an amelioration of the altered circulating cytokine profile in patients with IPAH, and does not aggravate the inflammatory state. The single bout of exercise in IPAH patients seems to be safe from an immune and inflammatory point of view.

## Abbreviations

6MWD: 6 min walk distance
ATS: American Thoracic Society
BMI: Body mass index
CCL: C-C motif ligand
CCR: C-C motif receptor
CD: Cluster of differentiation
CI: Cardiac index
CPET: Cardiopulmonary exercise testing
CRP: C-reactive Protein
CXCL: C-X-C motif ligand
CXCR: C-X-C motif receptor
ELISA: Enzyme linked immunosorbent assay
ERA: Endothelin receptor antagonists
ERS: European Respiratory Society
FACS: Fluorescence-activated cell sorting
FEV: Forced expiratory volume
FVC: Forced vital capacity
HPAH: Hereditary pulmonary arterial hypertension
IL: Interleukin
IPAH: Idiopathic pulmonary arterial hypertension
mPAP: Mean pulmonary arterial pressure
NT-proBNP: N-terminal pro-hormone of brain natriuretic peptide
PAH: Pulmonary arterial hypertension
PAWP: Pulmonary artery wedge pressure
PDE5-I: Phosphodiesterase type 5 inhibitor
PFT: Pulmonary function test
PH: Pulmonary hypertension
PVR: Pulmonary vascular resistance
rANOVA: Repeated analysis of variances
RAP: Right atrial pressure
sGC-S: Soluble guanylate cyclase stimulator
TCR: T cell receptor
Th: T helper
TNF: Tumor necrosis factor
WHO-FC: World Health Organization functional class
